# The Contemporary Role of Virtual and Augmented Reality in Radiation Oncology: A Review

**DOI:** 10.3390/curroncol33050279

**Published:** 2026-05-09

**Authors:** Saad Mohssine, Marie-Ève Pelland, Stephane Bedwani, David Roberge

**Affiliations:** 1 Division of Radiation Oncology, University of Montreal Hospital Center (CHUM), Montreal, QC H2X 0C1, Canada; 2 Department of Radiology, Radiation Oncology and Nuclear Medicine, Faculty of Medicine, Université de Montréal, Montreal, QC H3T 1J4, Canada

**Keywords:** virtual reality, augmented reality, radiation oncology, medical education, patient anxiety, radiotherapy workflow, immersive technologies, clinical innovation

## Abstract

**Purpose:** Virtual reality (VR) and augmented reality (AR) are increasingly being explored as tools with potential applications in radiation oncology, with applications in education, patient care, and workflow optimization. This review evaluates the current landscape and future potential of immersive technologies in clinical practice. **Methods:** A comprehensive literature review was conducted via PubMed and Scopus (search date: January 2026) using a Boolean search strategy combining terms for virtual/augmented/mixed reality, radiotherapy, and education/anxiety/training; articles published between 2010 and 2026 were screened independently by two reviewers. An online survey assessed experience with and perceptions of VR among Canadian radiation oncologists. Semi-structured interviews with physicians, residents, therapists, physicists, and a staff psychologist at a large academic tertiary-care center in Canada explored qualitative insights into current use and attitudes toward immersive technologies. **Results:** VR and AR show utility across multiple domains. In education, platforms such as the Virtual Environment for Radiotherapy Training (VERT) enable therapists to practice in simulated treatment environments, while VR-based contouring tools reduce segmentation time by 41–58% and improve spatial understanding. For patient care, immersive VR interventions reduce pre-treatment anxiety by 26–56%, enhance understanding of procedures, and may decrease sedation in pediatric populations. AR-guided positioning systems demonstrate feasibility with acceptable accuracy, offering radiation-free setup verification. Survey findings revealed limited adoption (>80% reported no use), though 40% believed VR could enhance patient education and 39% desired expanded integration over the next decade. Barriers included cost, limited institutional awareness, and lack of training infrastructure. **Conclusions:** VR and AR show early potential for improving education, reducing patient anxiety, and enhancing positioning accuracy in radiation oncology. Despite implementation barriers, ongoing trials and technological advances are gradually building the evidence needed to clarify the role of immersive technologies in clinical practice.

## Simple Summary

Cancer treatment using radiation, known as radiotherapy, can be a significant source of fear and anxiety for patients who are unfamiliar with the machines and procedures involved. Healthcare professionals who deliver these treatments also require specialized training that can be difficult to access. This review examines how virtual reality—a technology that immerses users in a computer-generated environment—and augmented reality—which overlays digital images onto the real world—are being used in radiation therapy. By analyzing the published scientific literature, conducting a national survey of Canadian radiation therapy professionals, and interviewing staff at a major Canadian cancer center, we found that these immersive technologies show early promise for reducing patient anxiety before and during treatment, improve the training of healthcare providers through realistic simulations, and enhance the accuracy of patient positioning during therapy. Despite considerable enthusiasm, more than 80% of surveyed Canadian professionals reported no active use of these technologies, citing cost, limited awareness, and lack of supporting infrastructure as the main barriers. As these technologies become more affordable and accessible, further rigorous research will be needed to confirm their benefit for cancer patients and the healthcare professionals who care for them.

## 1. Introduction

Immersive and interactive technologies hold growing potential to transform how we entertain, learn, communicate, and deliver services across various fields, including healthcare. Among these innovations, augmented reality (AR) and virtual reality (VR) are emerging as powerful healthcare tools with the potential to improve both clinical practice and patient experience. Virtual reality creates immersive, computer-generated environments in which users are isolated from their physical surroundings. In contrast, augmented reality overlays digital elements onto the real-world environment in real time, allowing users to remain aware of and interact with their physical setting [1,2]. In medicine, more broadly, these technologies have been sparsely adopted for surgical planning, medical education, and enhancing patient engagement [3].

Within radiation oncology, a specialty that demands spatial precision, workflow efficiency, and clear communication, VR and AR are being explored for diverse applications. These include patient and staff education, treatment planning, and management of patient compliance [4,5,6]. If properly implemented, these technologies have the potential to strengthen collaborative care by improving patients’ understanding of medical procedures.

Despite the long-standing use of complex software, advanced imaging and vision-based surface guidance, radiotherapy applications of VR and AR only began to appear in the literature in the early 2000s. Consumer VR and AR technologies have since improved significantly. Advancements in artificial intelligence and hardware technologies have improved performance, accessibility, and affordability.

Devices such as the Meta Quest 3 (Meta Platforms, Inc., Menlo Park, CA, USA), HTC Vive (HTC Corporation, New Taipei City, Taiwan), and Microsoft HoloLens 2 (Microsoft Corporation, Redmond, WA, USA) now offer high-resolution, user-friendly platforms suitable for everybody, including physicians, radiation therapists, students, and even patients [7]. These devices are head-mounted headsets that can immerse the user in a fully virtual environment by blocking out the physical world. The user can then, through motion tracking and handheld controllers, navigate and interact with 3D content in real time. For AR, similar hardware is used; however, instead of replacing the real world, digital content is superimposed onto it, allowing users to remain engaged with their physical surroundings. Importantly, many of today’s devices no longer require connection to a high-performance computer or the setup of external sensors/cameras in the room, making them more portable and easier to deploy in clinical or educational settings.

This review examines the current landscape and future potential of VR and AR in radiation oncology. We first describe how these technologies are applied in training and education before turning to their role in patient care, workflow optimization, and privacy protection.

## 2. Methods

### 2.1. Literature Search Strategy

A multi-staged literature review was conducted to evaluate the role of immersive technologies in healthcare, with a specific focus on radiation oncology. Systematic searches were performed across the PubMed (https://pubmed.ncbi.nlm.nih.gov/, accessed on 15 January 2026) and Scopus (https://www.scopus.com/, accessed on 15 January 2026) databases to capture the landscape of virtual reality (VR), and augmented reality (AR). The targeted analysis utilized a comprehensive Boolean string: (“Virtual Reality” OR “Augmented Reality”) AND (“Radiotherapy” OR “Radiation Oncology”) AND (“Medical Education” OR “Patient Education” OR “Anxiety” OR “Distress” OR “Training”). This search included peer-reviewed articles published between 2010 and 2026. While the parameters spanned sixteen years, the majority of included studies were published from 2020 onwards, reflecting the accelerating technological changes and increased interest in clinical integration. Additional studies were identified through manual citation tracking of the retrieved literature to ensure comprehensive coverage.

To identify ongoing, recently completed, or unpublished clinical trials, we conducted an additional search of the ClinicalTrials.gov (https://clinicaltrials.gov/) registry in April 2026. The search strategy utilized combinations of the following keywords: ‘VR’, ‘AR’, ‘Radiation Therapy’, and ‘Anxiety’. No restrictions were placed on the study start date or geographical location to ensure a comprehensive overview of the field.

### 2.2. Study Selection and Inclusion Criteria

**Figure 1:**
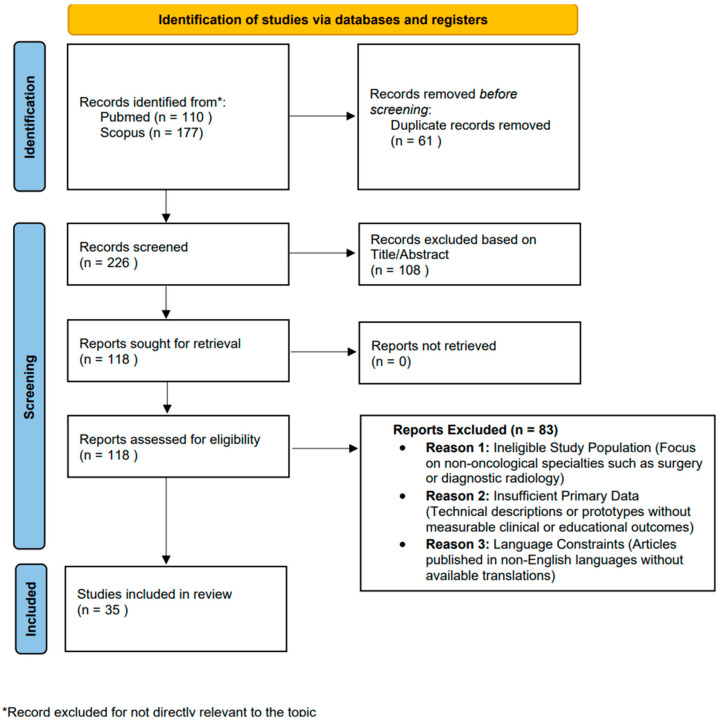
PRISMA flow diagram.

Studies were included if they demonstrated a primary focus on VR or ARtechnologies specifically within the field of radiation oncology. Furthermore, articles were required to evaluate specific outcomes. The systematic selection process, including the identification, screening, and specific reasons for exclusion at each stage, is visually summarized in the PRISMA-like flow diagram (Figure 1). As illustrated in the diagram, records were excluded based on three primary justifications. The first reason for exclusion was an ineligible study population, specifically focusing on non-oncological specialties such as general surgery or diagnostic radiology. The second reason involved insufficient primary data, where articles provided technical descriptions or prototypes without reporting measurable clinical or educational outcomes. Finally, articles were excluded due to language constraints, specifically those published in other non-English languages where no official translation was available for synthesis. This process resulted in a final library of 35 peer-reviewed articles for analysis. Screening of titles and abstracts was performed independently by two reviewers (S.M. and D.R.), with discrepancies resolved through discussion and consensus. Full-text articles were subsequently assessed for eligibility by the same two reviewers. Given the heterogeneous and predominantly descriptive nature of the included studies, a formal risk-of-bias assessment using a standardized tool was not applicable; however, methodological quality was considered informally during data synthesis, with attention to study design, sample size, outcome reporting, and potential sources of bias noted narratively where relevant.

### 2.3. Qualitative Interviews

Semi-structured interviews were conducted at the Centre Hospitalier de l’Université de Montréal (CHUM) to gather multidisciplinary insights. The participant cohort (*n* = 23) consisted of 3 physicians, 6 radiation oncology residents, 5 radiation oncologists, 8 radiation therapists, and 1 staff psychologist. Participants were selected via purposive sampling to ensure representation across all key clinical roles in the radiotherapy workflow. To maintain transparency and accuracy without audio recording, detailed written notes were taken during each session and reviewed immediately afterward for completeness. Thematic analysis was employed to synthesize this qualitative feedback, with recurring motifs regarding the benefits and challenges of clinical VR/AR adoption identified through a collaborative review of the records by the research team. This process focused on identifying institutional attitudes and the perceived psychological impact of immersive technologies on patient anxiety management. Data collection was guided by the principle of thematic saturation, which was reached when subsequent interviews with the multidisciplinary staff yielded no new insights or recurring motifs, confirming that the *n* = 18 sample was sufficient to capture the full range of institutional perspectives.

### 2.4. National Survey

To assess professional perceptions and current utilization across Canada, an online survey was distributed through the Canadian Association of Radiation Oncology (CARO). A formal institutional ethics waiver was obtained prior to the distribution of the survey. The questionnaire was structured into four key dimensions: (1) current individual and institutional utilization of immersive tools; (2) perceived clinical and educational potential; (3) institutional barriers evaluated through 5-point Likert scales; and (4) professional interest in future training and development.

The survey also included optional qualitative sections to capture narrative feedback on previous clinical experiences and strategic recommendations for integrating these technologies within the Canadian landscape.

## 3. Results

### 3.1. Virtual and Augmented Reality in Radiation Oncology

#### Education

Simulation-based technologies are effective in the education and training of healthcare professionals, as they replicate real-life clinical scenarios within a controlled immersive environment. This approach allows trainees to gain exposure to diverse clinical situations while developing relevant technical and cognitive skills. Recent publications have claimed benefits across a wide range of healthcare disciplines, including nursing, physiotherapy and medicine—providing tailored learning opportunities for students and practicing professionals alike [8]. For example, in emergency medicine, virtual reality simulations can recreate high-pressure scenarios such as cardiac arrest, allowing trainees to practice critical decision-making, teamwork, and resuscitation techniques without risk to real patients [9]. Moreover, VR training is often more efficient than traditional simulation labs, which require dedicated rooms with clinical simulation mannequins and the presence of multiple instructors to provide real-time feedback. Other examples include surgical trainees using virtual simulations to practice laparoscopic techniques in simulated operating room environments [10]. These technologies offer the flexibility of self-directed learning, as most simulations can be accessed independently without the need for constant supervision. Moreover, many VR platforms provide feedback through the headset, offering real-time guidance on performance and helping learners correct errors during training. This often includes haptic feedback, a virtual reality component that delivers tactile sensations such as vibration or resistance through controllers or wearable devices. For example, HapticVR (FundamentalVR, Boston, MA, USA), a surgical simulation platform, uses haptic feedback combined with performance analytics to provide immediate insights into metrics such as precision, applied force, and task completion time, thereby supporting skill acquisition through adaptive feedback [11].

Residency training has traditionally relied on a combination of classroom-based instruction, textbook learning, supervised use of treatment planning software, and hands-on experience in clinical settings. Radiation oncology residents typically learn tasks such as contouring and dosimetry by working within treatment planning systems, often under faculty supervision. In contrast, radiation therapists are trained in machine operation and daily treatment delivery using devices dedicated to learning and/or installed in a clinical environment, gaining proficiency through in-clinic observation and supervised practice. Both groups face challenges including the changing landscape of radiotherapy, limited access to expensive equipment, busy clinical schedules, and concerns about patient safety. Virtual reality is increasingly being explored as a complementary educational tool. Platforms such as the Virtual Environment for Radiotherapy Training (VERT) simulate radiotherapy treatment rooms, enabling radiation therapists to familiarize themselves with the spatial relationships between the patient, equipment, and beam arrangements in a safe and interactive setting. This technology typically projects a simulation of a radiotherapy treatment bunker—complete with a linear accelerator—onto a screen (with optional 3D glasses), allowing users to practice controlling the treatment couch and machine components using controllers or hand gestures [12]. Such VR tools can increase training capacity for therapists, providing hands-on experience without occupying clinical resources. Additionally, they enhance the learning process by transforming traditional instruction into immersive, three-dimensional experiences that improve understanding and retention [13]. Importantly, VR training systems offer a cost-effective alternative for educational institutions, being far less expensive than purchasing and maintaining a full linear accelerator for training purposes. VR also has the potential of simulating a broad range of radiotherapy devices.

Another key educational use of virtual reality in radiation oncology education lies in the teaching of dosimetry and contouring, which require a good understanding of spatial relationships between tumors, organs at risk, and dose. Traditional 2D CT-slice viewing can limit deep understanding, whereas VR platforms provide a potentially more informative 3D perspective. Virtual reality platforms such as Realize Medical (RealizeMedical, Ottawa, ON, Canada) enable users to interact with three-dimensional reconstructions of patient anatomy, allowing for real-time manipulation and exploration of target volumes, isodose curves, and beam paths in an immersive environment. This approach has been reported to improve contouring accuracy and inter-observer consistency among trainees by providing a more intuitive spatial understanding of radiologic anatomy and treatment objectives. According to a study conducted by Belec et al. (2024), VR-based contouring reduced the time required to contour complex cranial and spinal target by an average of 41% for a junior radiation oncology resident and 58% for an experienced radiation oncologist [14]. This emphasizes the potential of VR in segmentation. Platforms like VRContour, developed by Chen et al. (2022) [15], further extend this functionality by importing DICOM RT structure sets and CT datasets directly into a virtual reality interface, enabling users to visualize and edit structures from multiple perspectives with enhanced depth perception. Notably, VRContour has been reported to improve contouring performance by allowing users to identify inconsistencies in anatomical boundaries that are often missed in standard 2D views. In their pilot evaluation, users reported that working within VR provided a clearer sense of spatial relationships between organs and improved their confidence in contouring tasks—benefits particularly valuable for trainees and for interdisciplinary collaboration [15,16,17].

Virtual reality is also emerging as a tool to improve education and procedural readiness in brachytherapy, particularly in gynecologic oncology. Brachytherapy procedures require hands-on experience to develop spatial awareness of internal pelvic anatomy, an understanding of dose distribution, and the ability to perform under time-sensitive pressured conditions. Traditional training methods, such as direct observation, supervised clinical procedures, and scant access to cadaver-based workshops, are limited by case availability, variability in teaching, and a lack of standardized feedback. VR-based simulations have the potential to address these limitations by providing a consistent, reproducible environment where learners can rehearse a range of procedures from start to finish.

In a randomized study, medical residents using VR platforms to train in gynecologic brachytherapy demonstrated significant improvements in technical proficiency, procedural confidence, and engagement compared to those receiving conventional instruction [18]. These simulations allowed users to practice applicator insertions, visualize three-dimensional dose distributions, and receive real-time performance feedback, enhancing both their psychomotor and decision-making skills. Furthermore, case reports have shown that VR-guided simulations can also be tailored for preoperative planning in complex cases, offering clinicians a tool to anticipate anatomical challenges and refine their approach before entering the operating room [19,20]. As these systems become more accessible and anatomically accurate, integrating VR into residency curricula and ongoing professional development may reduce the learning curve, minimize procedural errors, and ultimately improve the safety and efficacy of brachytherapy.

### 3.2. Patient Education and Anxiety Management

Transitioning from training to patient-facing applications, VR and AR are increasingly explored as adjuncts to improve the patient experience in radiation oncology. Undergoing radiotherapy is frequently associated with psychological distress, particularly among individuals receiving cancer treatment for the first time. Anxiety may occur before and during radiotherapy treatment due to uncertainty about procedures, concerns about side effects, the imposing appearance of treatment equipment, and the emotional impact of a cancer diagnosis. Claustrophobia (defined as a persistent fear of confined spaces) can further exacerbate distress and lead to panic or avoidance behaviors in settings that require immobilization or confinement, such as MRI imaging or head and neck radiotherapy.

Anxiety and claustrophobia have traditionally been managed through patient education, relaxation techniques, pharmacotherapy and psychological support. Patients are often referred for desensitization programs that include cognitive behavioral therapy, breathing exercises, and progressive exposure to the treatment environment [21]. These programs may require multiple sessions over several weeks. Although effective for many patients, these interventions are resource-intensive, may delay radiotherapy and will not fully alleviate distress in all cases. Structured pre-procedure education delivered by trained clinicians has been identified as a leading strategy to facilitate completion of imaging and treatment sessions; for example, supportive discussion prior to MRI has been reported as more effective than several alternative behavioral or technical approaches [22].

Of note, head and neck cancer patients represent a subgroup at heightened risk for treatment related anxiety. Thermoplastic masks can be both physically uncomfortable and psychologically distressing. Some patients report the onset of anxiety only after the mask is tightly secured to the treatment table, eliciting feelings of confinement and helplessness [23]. Targeted preparation and expectation setting are therefore critical in this population.

Traditional methods of patient preparation, such as verbal explanations and printed material, may fall short in fully addressing these emotional and informational needs. As a result, improving patient education and anxiety management is a growing focus in radiation oncology, with emerging technologies such as VR and AR offering new approaches to enhance understanding, reduce fear, and promote a more positive treatment experience.

Recent advances in virtual and augmented reality offer immersive, visual tools that may complement established strategies to patient education and anxiety management. Immersive VR simulations, such as those delivered via Panosphere 360 (Panoreality, Boucherville, QC, Canada), allow patients to explore a simulated treatment environment prior to therapy, with the goal of demystifying procedures and improving situational familiarity. In a pilot randomized trial, VR-based education was associated with reductions in self-reported anxiety, as measured by validated scales and improvements in selected physiological markers such as blood pressure. Participants also demonstrated greater understanding of treatment procedures compared to those receiving standard pre-treatment information [24]. Similarly, a systematic review of interventional studies involving 376 adults across multiple countries confirmed that VR-enhanced educational sessions improved patient knowledge of radiotherapy and lowered pre-treatment anxiety overall [25].

In practical terms, standalone handheld headsets enable patients to virtually navigate the treatment vault, observe life-sized representations of themselves on the treatment couch, and visualize radiotherapy beam delivery.

Department level initiatives have used panoramic video to provide pre-treatment tours, with the objective of clarifying the sequence of care and reducing uncertainty. A notable example is Penn Medicine’s Radiation Oncology department, which offered VR tours of their treatment center using Google Cardboard-compatible videos to guide patients through radiotherapy process. This initiative aims to improve understanding and reduce anxiety by providing clear visual explanations of what to expect before the first session [26]. Patients surveyed reported feeling “like they were truly in the treatment room”, gaining clarity on how beams intersect and why immobilization masks are needed. After the VR session, 93% of participants indicated improved understanding of their treatment, and of those with baseline anxiety, 57% reported reduced fear following the VR experience [27].

Augmented reality tools also hold significant promises by enabling smartphones or headsets to project holographic representations of the treatment machine or patient anatomy during medical consultation. These visualizations can facilitate interactive discussions, help clarify complex procedural steps, and support shared decision-making in real time [28]. Collectively, such immersive technologies enhance patient preparedness, reduce emotional distress, and foster a greater sense of trust and engagement. While clinical evidence remains preliminary, AR may further enhance preparedness and engagement, particularly for patients at higher risk of anxiety or claustrophobia.

### 3.3. Patient Positioning

Proper patient positioning is important to effective and efficient radiotherapy. Improper initial positioning can mean longer and less accurate treatment sessions. Conventionally, initial patient setup relies on laser alignment, physical immobilization devices, and increasingly, surface guidance with structured light [29]. Recent advances in augmented reality have opened new avenues for enhancing positioning speed and accuracy. Improved initial positioning may reduce radiation dose from repeat setup imaging. Several studies have explored the development of AR-assisted positioning systems. While a standardized patient positioning system is yet to be fully developed for widespread clinical use, the referenced studies describe clinical prototypes which superimpose virtual data onto the patient’s body in real time, providing radiation therapists with intuitive visual guidance. Augmented reality positioning systems typically employ head-mounted displays or tablet-based platforms to overlay reference images or point clouds on the patient surface.

The Zhang et al. [30] study demonstrated that AR-assisted radiotherapy positioning is feasible with “acceptable” accuracy compared to traditional methods. In their phantom-based evaluation, the anthropomorphic phantom positioning errors were 3.1 ± 1.9 mm, 2.4 ± 2.5 mm, and 4.6 ± 2.8 mm in the lateral, vertical, and longitudinal axes respectively, with rotational deviation of 0.26 ± 0.14°. The study used HoloLens 2 and 3D holographic models reconstructed from simulation CT images to guide patient positioning. Similarly, a clinical evaluation by Li et al. [31] further quantified these benefits using an AR-guided positioning system (ARPT) based on 3D reconstructions from simulation CT data and point cloud registration. In a study of 40 patients, the ARPT system significantly improved accuracy for truncal sites compared to conventional laser-based methods. For patients with chest and abdomen cancer, residual errors were significantly reduced in the superior–inferior (1.41 ± 0.83 mm vs. 3.13 ± 0.85 mm; t = −5.80, *p* < 0.001) and anterior–posterior directions (1.64 ± 0.96 mm vs. 3.20 ± 1.86 mm; t = −4.98, *p* < 0.001). Statistically significant improvements were also observed across all three rotational directions (*p* < 0.05), effectively reducing the frequency of superior–inferior translational errors exceeding 5 mm from 16.7% to 0%. However, in the head and neck cohort, the difference between the two positioning methods was not statistically significant (*p* > 0.05). These findings demonstrate that AR technology can be utilized in the positioning of patients with truncal cancers, reducing setup errors in the chest and abdomen thus potentially improving treatment accuracy.

Another study presented an augmented reality-guided radiotherapy position system that incorporates point cloud registration to enable accurate and markerless patient setup. Using an iPad Pro (Apple Inc., Cupertino, CA, USA) equipped with a LiDAR camera, the system captures real-time 3D surface data from the patient and aligns it with the planning CT using iterative closest point algorithms. This approach allows AR projections of the patient’s contours or body surface to be superimposed directly onto the treatment couch in real time. The technique demonstrated high positioning precision, with mean translational errors below 1 mm and angular errors under 1°. It maintained performance under varying lighting conditions and minor patient movement. Its contactless operation enhances patient comfort, while the portability and affordability of AR devices based on consumer electronics may facilitate broader integration and simplify user training [32].

### 3.4. Qualitative Interviews

Semi-structured interviews conducted with multidisciplinary staff at the Centre Hospitalier de l’Université de Montréal (CHUM) revealed four primary themes regarding immersive technology integration. First, regarding patient psychology, the staff psychologist and radiation therapists noted that immersive VR tours could proactively demystify the treatment vault, potentially reducing the claustrophobia and anticipatory anxiety associated with immobilization devices, such as thermoplastic masks. Second, regarding medical training, physicians and residents expressed strong interest in VR contouring tools, emphasizing their potential to convey complex 3D spatial relationships compared to traditional 2D monitors. Third, regarding technical integration, medical physicists emphasized the critical need for the rigorous and safe commissioning of new technologies, the establishment of robust quality assurance processes, and the development of clinical workflows that seamlessly integrate into the broader radiation oncology ecosystem. Finally, regarding implementation barriers, participants consistently cited the initial learning curve for staff and the logistical challenges of managing and sanitizing headsets between patient uses as hurdles to daily clinical integration.

### 3.5. National Survey Findings

To evaluate current institutional awareness and utilization across Canada, a national survey was distributed to approximately 330 radiation oncology professionals, yielding 34 responses (10.3% response rate). While this response rate indicates a potential for non-response bias, the data provides useful insights into the Canadian landscape. Current adoption of immersive technologies remains profoundly limited, with more than 80% of respondents reporting no active use of VR or AR in their clinical or educational practice. Despite this low baseline utilization, professional perception of the technology’s utility was notably high; quantitative analysis revealed that 58% of participants agreed (40%) or strongly agreed (18%) that VR could significantly enhance patient understanding of the treatment process by simulating the radiotherapy environment. Additionally, 39% of respondents expressed a desire for VR to play a larger role in radiation oncology departments over the next decade, though many remained unsure, reflecting the current limited visibility of these tools in Canadian cancer centers. Qualitative insights corroborated these trends, with one respondent highlighting a successful application where VR deployment enabled pediatric patients to undergo radiotherapy without the need for systemic sedation. Across all responses, the barriers identified were financial limitations and a general lack of institutional awareness. These findings suggest that while the field is in its nascent stages, there is a developing consensus regarding the potential of VR to improve patient experience (provided that funding, accessibility, and training infrastructure are improved).

### 3.6. Clinical Utility and Implementation Barriers

Despite the high level of interest identified in both the literature and the national survey, several systemic hurdles currently prevent the widespread adoption of immersive technologies in clinical radiation oncology. These implementation barriers are categorized and summarized in Table 1, alongside the corresponding benefits and documented applications of these tools. As shown in the table, financial constraints, including the high upfront costs of hardware acquisition and specialized software development, remain the most significant deterrent for many institutions. Furthermore, technical limitations such as the lack of integration within existing Treatment Planning Systems (TPS) and the lack of standardized training protocols for clinical staff create operational friction. Addressing these challenges will be essential to transition VR and AR from experimental and niche academic settings into routine daily practice.

## 4. Discussion

The findings of this review and the accompanying national survey reveal a significant awareness gap within the Canadian radiation oncology landscape. While 58% of surveyed professionals acknowledge the potential for immersive technologies to demystify the treatment process—corroborating our local multidisciplinary insights regarding anxiety management—this positive perception has not yet translated into clinical adoption, with over 80% of respondents reporting no active use. This discrepancy suggests that while the clinical pull for VR and AR is strong, the structural pull is currently insufficient. Our findings align with international trends indicating that immersive tools are effective in reducing claustrophobia and anticipatory distress; however, their use remains largely confined to niche academic and pilot settings rather than standard clinical practice.

**Figure 2:**
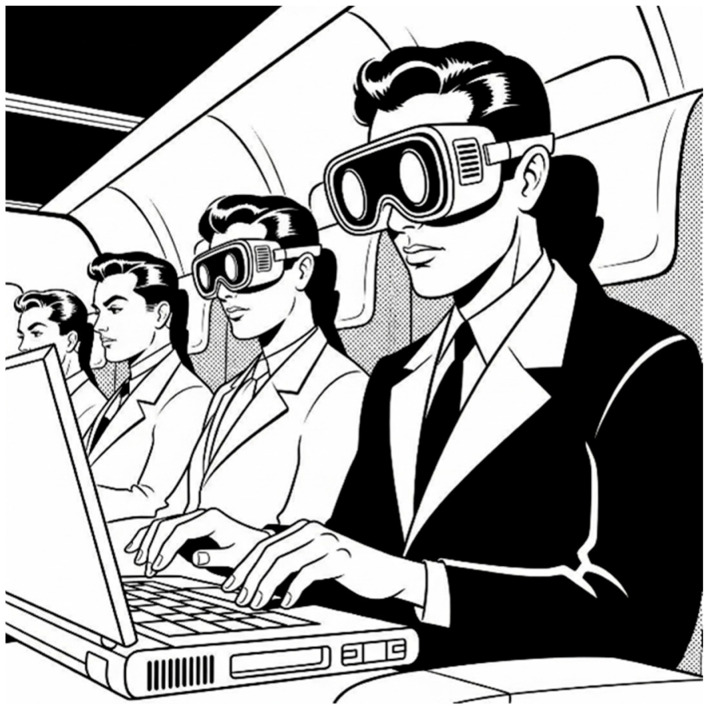
Stylized physician wearing a head-mounted display aboard an airplane, confidentially working in patient charts via his VR/AR interfaces. Image generated using Google Gemini AI (version 3.1 Pro, Google DeepMind, Mountain View, CA, USA; https://gemini.google.com/, accessed on 10 March 2026).

The integration of augmented reality (AR) into radiation oncology presents a significant opportunity to address emerging challenges in confidential data access, particularly as clinical workflows increasingly shift toward remote or non-traditional environments. While not yet a component of standard practice, AR-enabled smart glasses offer a theoretical framework for the private display of sensitive patient information, potentially facilitating secure charting, treatment planning, and real-time decision-making (Figure 2). Theoretical frameworks and interviewee vision suggest that such integrated applications could potentially enhance workflow efficiency, provided that technical and regulatory issues are addressed [33]. However, the transition from niche application to clinical adoption remains contingent upon rigorous evaluations of data security and regulatory compliance. Future implementation will require seamless integration with existing electronic health record (EHR) systems and comprehensive user training to ensure that patient confidentiality is robustly maintained across all care settings [34].

To bridge the gap between perceived utility and clinical reality, the systemic barriers identified in Table 1 must be addressed through a multifaceted approach. The financial constraints cited by respondents—specifically the high costs of specialized software and hardware maintenance—suggest that a “one-size-fits-all” high-fidelity VR approach may not be feasible for all centers. Instead, a tiered implementation strategy may be more effective, utilizing low-cost mobile VR platforms for basic patient education while reserving high-fidelity systems for specialized residency training and complex procedural simulation. Furthermore, the lack of institutional awareness highlights a need for task forces and guidelines. Peer-to-peer training and centralized resource sharing through organizations such as CARO could reduce the barriers which currently deters departments from adopting these tools.

Looking ahead, ongoing clinical trials are investigating the feasibility and efficacy of VR and AR technologies in radiation oncology. Notably, trial NCT04141943 examines whether delivering radiotherapy information through VR can enhance medical staff productivity, improve patient experience and satisfaction, as well as reduce anxiety prior to treatment [35]. Another study, NCT05947045, assesses the feasibility and acceptability of VR-based cognitive training for children undergoing radiotherapy for brain tumors, compared to tablet-based interventions [36]. A summary of these and other ongoing trials is presented in Table 2. The results of these studies will hopefully provide valuable evidence on the clinical utility of immersive technologies, advancing our understanding of their impact on both patient-centered outcomes and professional education.

This study is limited by the modest response rate of the national survey (10.3%), which may introduce non-response bias. Additionally, as the field of immersive technology is rapidly evolving, studies published during the final stages of this review may not be fully represented. Future research should prioritize multi-center longitudinal trials to quantify the long-term cost-effectiveness and clinical outcomes associated with these tools.

## 5. Conclusions

Virtual and augmented reality have the potential to advance radiation oncology practice. Although challenges to adoption and integration persist, emerging evidence and ongoing research continue to shed light on their clinical value. The future remains open, with the full impact of these technologies yet to be revealed.

## Figures and Tables

**Table 1:** Summary of VR/AR Applications in Radiation Oncology.

Application	Key Benefits	Limitations/Barriers
Patient Education & Treatment Preparation	• Enhances understanding of procedures • Reduces anxiety and claustrophobia • Improves engagement and satisfaction • May reduce need for sedation	• High upfront cost • Limited availability of VR modules • Requires staff training for facilitation
Simulation-Based Training (Residents & Therapy students)	• Safe hands-on practice • Improves contouring accuracy and procedural confidence • Allows rehearsal of rare/complex cases • Standardizes training across staff	• Hardware and software investment required • May not fully replicate real-world workflow pressures • Staff acceptance and adaptation vary
Contour & Dosimetry Education	• Provides 3D spatial understanding of anatomy • Reduces inter-observer variability • Enhances confidence in planning and treatment decisions	• Learning curve for software use • Limited integration with clinical planning systems
Brachytherapy Procedural Training	• Enables repeated, standardized practice • Improves procedural confidence and accuracy • Reduces dependency on cadaver labs or limited case availability	• Limited procedural modules • VR may not fully replicate tactile feedback in some procedures
Patient Positioning & AR Guidance	• Enhances accuracy of setup • Reduces inter-operator variability • Decreases repeated imaging and radiation exposure • Supports real-time decision-making	• Complex integration with existing equipment • Accuracy dependent on device calibration and environment • Requires staff training

**Table 2:** Ongoing clinical trials of VR and AR in radiation oncology, including NCT numbers, study title, and interventions.

NCT Number	Study Title	Interventions
NCT04934293	Virtual Reality for Children in Radiotherapy (REVER)	Virtual reality, Relaxation techniques, Digital sedation, Anxiety management
NCT06506435	Radiotherapy Patient Education with Virtual Reality	2D informational video, First-Person VR Video, Third-Person VR video, Patient experience
NCT05947045	Cognitive Training in the VR Setting With Children Undergoing Radiotherapy for Brain Tumors	Pediatric cognitive training
NCT06199050	The Effect of 360° Virtual Reality Movies on Fear and Anxiety	Anxiety management, Fear management, VR
NCT05440760	Using Virtual Reality Technology to Improve Patient Experience and Quality of Care During Brachytherapy	Patient experience during brachytherapy, VR distractions, Anxiety management
NCT06985784	VR Interventions for the Improvement of Depression, Anxiety and Pain in Patients With Head and Neck Cancer and Caregivers	Reducing depressive symptoms, anxiety management, Heart rate tracking

## Data Availability

The original contributions presented in this study are included in the article. Further inquiries can be directed to the corresponding authors.

## References

[1] Kok D.L., Dushyanthen S., Peters G., Sapkaroski D., Barrett M., Sim J., Eriksen J.G. (2022). Virtual reality and augmented reality in radiation oncology education—A review and expert commentary. Tech. Innov. Patient Support Radiat. Oncol..

[2] Eckert M., Volmerg J.S., Friedrich C.M. (2019). Augmented Reality in Medicine: Systematic and Bibliographic Review. JMIR Mhealth Uhealth.

[3] Bruno R.R., Wolff G., Wernly B., Masyuk M., Piayda K., Leaver S., Erkens R., Oehler D., Afzal S., Heidari H. (2022). Virtual and augmented reality in critical care medicine: The patient’s, clinician’s, and researcher’s perspective. Crit. Care.

[4] Cheung E.Y.W., Law M.Y.Y., Cheung F. (2021). The Role of Virtual Environment for Radiotherapy Training (VERT) in Medical Dosimetry Education. J. Cancer Educ. Off. J. Am. Assoc. Cancer Educ..

[5] van der Kruk S.R., Zielinski R., MacDougall H., Hughes-Barton D., Gunn K.M. (2022). Virtual reality as a patient education tool in healthcare: A scoping review. Patient Educ. Couns..

[6] Chen J.J., Brown A.M., Garda A.E., Kim E., McAvoy S.A., Perni S., Rooney M.K., Shiue K., Tonning K.L., Warren L.E. (2024). Patient Education Practices and Preferences of Radiation Oncologists and Interprofessional Radiation Therapy Care Teams: A Mixed-Methods Study Exploring Strategies for Effective Patient Education Delivery. Int. J. Radiat. Oncol. Biol. Phys..

[7] Barsom E.Z., Graafland M., Schijven M.P. (2016). Systematic review on the effectiveness of augmented reality applications in medical training. Surg. Endosc..

[8] Lateef F. (2010). Simulation-based learning: Just like the real thing. J. Emerg. Trauma Shock.

[9] Mahling M., Wunderlich R., Steiner D., Gorgati E., Festl-Wietek T., Herrmann-Werner A. (2023). Virtual Reality for Emergency Medicine Training in Medical School: Prospective, Large-Cohort Implementation Study. J. Med. Internet Res..

[10] Portelli M., Bianco S.F., Bezzina T., Abela J.E. (2020). Virtual reality training compared with apprenticeship training in laparoscopic surgery: A meta-analysis. Ann. R. Coll. Surg. Engl..

[11] Fundamental XR Immersive Technology to Accelerate Human Excellence—Solutions in Vr, Ar, And Mixed Reality for Healthcare Training and Simulation. https://www.fundamentalxr.com/.

[12] Ramashia P.N. (2023). Radiotherapy plan evaluation tool in a resource-limited setting: Comparison of VERT and treatment planning software. J. Med. Imaging Radiat. Sci..

[13] Boejen A., Grau C. (2011). Virtual reality in radiation therapy training. Surg. Oncol..

[14] Belec J., Sutherland J., Volpini M., Rakhra K., Granville D., La Russa D., Flaxman T., De Oliveira E.P., Glikstein R., Dos Santos M.P. (2024). A Pilot Clinical and Technical Validation of an Immersive Virtual Reality Platform for 3D Anatomical Modeling and Contouring in Support of Surgical and Radiation Oncology Treatment Planning. J. Imaging Inform. Med..

[15] Chen C., Yarmand M., Singh V., Sherer M.V., Murphy J.D., Zhang Y., Weibel N. VRContour: Bringing Contour Delineations of Medical Structures into Virtual Reality. Proceedings of the 2022 IEEE International Symposium on Mixed and Augmented Reality (ISMAR).

[16] Liu J., Li X., Leng X., Zhong B., Liu Y., Liu L. (2022). Effect of 3D Slicer Preoperative Planning and Intraoperative Guidance with Mobile Phone Virtual Reality Technology on Brain Glioma Surgery. Contrast Media Mol. Imaging.

[17] Rooney M.K., Zhu F., Gillespie E.F., Gunther J.R., McKillip R.P., Lineberry M., Tekian A., Golden D.W. (2018). Simulation as More Than a Treatment-Planning Tool: A Systematic Review of the Literature on Radiation Oncology Simulation-Based Medical Education. Int. J. Radiat. Oncol. Biol. Phys..

[18] Prabhu A.V., Peterman M., Kesaria A., Samanta S., Crownover R., Lewis G.D. (2023). Virtual reality technology: A potential tool to enhance brachytherapy training and delivery. Brachytherapy.

[19] Okuma K., Nakayama H., Sakuramachi M., Kimura S., Yatsuoka W., Yoshiba A., Inaba K., Kaneda T., Kashihara T., Takahashi K. (2025). Virtual reality-guided simulation of brachytherapy: Two case reports. J. Contemp. Brachytherapy.

[20] Taunk N.K., Shah N.K., Hubley E., Anamalayil S., Trotter J.W., Li T. (2021). Virtual reality-based simulation improves gynecologic brachytherapy proficiency, engagement, and trainee self-confidence. Brachytherapy.

[21] Carmack C.L., Agosta M.T., Ann-Yi S., Bruera E. (2023). Treating Radiation Anxiety with Systematic Desensitization: Head and Neck Cancer Case Reports. J. Palliat. Med..

[22] Al-Shemmari A.F., Herbland A., Akudjedu T.N., Lawal O. (2022). Radiographer’s confidence in managing patients with claustrophobia during magnetic resonance imaging. Radiography.

[23] Forbes E., Clover K., Baker A.L., Britton B., Carlson M., McCarter K. (2023). ‘Having the mask on didn’t worry me until…they clamped my head down so I wouldn’t move’: A qualitative study exploring anxiety in patients with head and neck cancer during radiation therapy. J. Med. Radiat. Sci..

[24] Wang L.J., Casto B., Reyes-Molyneux N., Chance W.W., Wang S.J. (2023). Smartphone-based augmented reality patient education in radiation oncology. Tech. Innov. Patient Support Radiat. Oncol..

[25] Grilo A.M., Almeida B., Rodrigues C., Isabel Gomes A., Caetano M. (2023). Using virtual reality to prepare patients for radiotherapy: A systematic review of interventional studies with educational sessions. Tech. Innov. Patient Support Radiat. Oncol..

[26] Taylor M. (2024). Penn Medicine uses virtual reality to ease radiation fears. Becker’s Hospital Review.

[27] Wang L.J., Casto B., Luh J.Y., Wang S.J. (2022). Virtual Reality-Based Education for Patients Undergoing Radiation Therapy. J. Cancer Educ. Off. J. Am. Assoc. Cancer Educ..

[28] Haleem A., Javaid M., Khan I.H. (2020). Holography applications toward medical field: An overview. Indian J. Radiol. Imaging.

[29] Volz L., Korte J., Martire M.C., Zhang Y., Hardcastle N., Durante M., Kron T., Graeff C. (2024). Opportunities and challenges of upright patient positioning in radiotherapy. Phys. Med. Biol..

[30] Zhang G., Liu X., Wang L., Zhu J., Yu J. (2022). Development and feasibility evaluation of an AR-assisted radiotherapy positioning system. Front. Oncol..

[31] Li C., Lu Z., He M., Sui J., Lin T., Xie K., Sun J., Ni X. (2022). Augmented reality-guided positioning system for radiotherapy patients. J. Appl. Clin. Med. Phys..

[32] Zhai S., Wei Z., Wu X., Xing L., Yu J., Qian J. (2024). Feasibility evaluation of radiotherapy positioning system guided by augmented reality and point cloud registration. J. Appl. Clin. Med. Phys..

[33] Niimi Y., Ota K. (2013). Display methods of electronic patient record screens: Patient privacy concerns. Stud. Health Technol. Inform..

[34] Ara J., Karim F.B., Alsubaie M.S.A., Bhuiyan Y.A., Bhuiyan M.I., Bhyan S.B., Bhuiyan H. (2021). Comprehensive Analysis of Augmented Reality Technology in Modern Healthcare System. Int. J. Adv. Comput. Sci. Appl. (IJACSA).

[35] ClinicalTrials.gov Effect of Virtual Reality (Vr) Information on Radiotherapy Anxiety and Medical Staff Productivity (Nct04141943). https://clinicaltrials.gov/ct2/show/NCT04141943.

[36] St. Jude Children’s Research Hospital (2023). Cognitive Training in the Virtual Reality Setting with Children Undergoing Radiotherapy for Brain Tumors (ClinicalTrials.gov Identifier: NCT05947045). ClinicalTrials.gov. NCT05947045.

